# The ocular biometry characteristics of young patients with primary angle-closure glaucoma

**DOI:** 10.1186/s12886-022-02374-2

**Published:** 2022-04-02

**Authors:** Yi Xu, Qian Tan, Chunyan Li, Dan Liu

**Affiliations:** 1 Eye Center of Xiangya Hospital, Central South University, 87 Xiangya Road, Changsha, China; 2 Hunan Key Laboratory of Ophthalmology, 87 Xiangya Road, Changsha, China; 3 Xiangya Hospital of National Geriatric Disease Clinical Medical Research Center, 87 Xiangya Road, Changsha, China

**Keywords:** Ocular biometric characteristics, Young patients, Primary angle-closure glaucoma

## Abstract

**Background:**

Although primary angle-closure glaucoma (PACG) mainly occurs in elderly people, diagnosis of PACG in young patients is not uncommon. So far, there is no article specialized on the ocular anatomical characteristics in these patients.In this study, ocular anatomical characteristics in young PACG patients are analyzed.

**Methods:**

In this retrospective, comparative study, patients diagnosed with PACG and received ultrasound biomicroscopy (UBM) examination in our department were included. Patients were divided into two groups: a young group composed of patients ≤ 45 years and an old group composed of patients > 45 years. A-scan ultrasonography and ultrasound biomicroscopy (UBM) were used to measure ocular biometric parameters of patients in the two groups including axial length (AL), lens thickness (LT), central anterior chamber depth (ACD), anterior chamber width (ACW), angle opening distance 500 (AOD500), anterior chamber angle 500 (ACA500), iris thickness 1000 μm from the iris root (IT1000), iris thickness 500 μm from the iris root (IT500), trabecular-ciliary process angle (TCPA), trabecular-ciliary process distance (TCPD), scleral– ciliary process angle (SCPA), lens vault (LV), and pupil diameter (PD). Plateau iris (PI) and basal iris insertion were determined from UBM images, and the prevalence of PI and basal iris insertion were compared between the two groups. The incidence of postoperative malignant glaucoma (MG) was also determined in both groups and ocular anatomical predictors for the development of MG were evaluated in young PACG patients.

**Results:**

One hundred fifteen patients were included into young group and 480 patients were included into old group. The young group had shorter TCPD, shorter AL, narrower TCPA, narrower SCPA thinner Lens compared to the old group. There were no significant differences in ACD, AOD 500, ACA500, LV, IT500, IT1000, PD or ACW between the two groups. The prevalence of PI was 22.6% in old group and 66.1% in young group (*P* < 0.001). More young PACG patients displayed basal iris insertion compared to old PACG patients (*P* < 0.001). 87 patients in the young group and 201 patients in the old group underwent trabeculectomy in our study. Among these patients, 21 young patients and 11 old patients developed MG after trabeculectomy (*P* < 0.001).

**Conclusions:**

Shorter AL, more anteriorly positioned ciliary body, higher prevalence of PI may be responsible for the etiology of young PACG patients. Our results suggest that shorter AL, shorter TCPD and narrower TCPA may be predictors for development of MG in young PACG patients after trabeculectomy.

## Introduction

Primary angle-closure glaucoma (PACG) is a major form of glaucoma in Asia, affecting approximately 0.75% Asian adults. Although the disease mainly occurs in elderly people, diagnosis of PACG in young patients is not uncommon [1, 2].

Besides some smaller case reports [3, 4], only one study, conducted by Ritch et al., systematically examined the demographics and clinical appearance of young individuals with angle closure [5]. Their investigation of angle closure in patients aged 40 years and younger found that the etiology of angle closure in young individuals is typically associated with structural developmental ocular anomalies rather than relative pupillary block; however, they did not evaluate ocular anatomical characteristics in these patients.

Eyes with PACG tend to have certain biometric characteristics including a shallow anterior chamber depth (ACD), a thick and anteriorly positioned lens, and a short axial length (AL) [6]. Since occlusion of the peripheral anterior chamber angle (ACA) is the primary cause of primary angle closure (PAC), the anatomic configurations around the peripheral ACA, including the iris root and the ciliary body, also play important roles in the development of PAC [7, 8]. In the present study, the ocular biometric parameters associated with PAC were compared between young and old patients with PACG. Although pupillary block is considered the most common cause of angle closure, non-pupillary block mechanisms, such as plateau iris (PI), may be responsible for a significant proportion of angle closure, especially in young patients [2, 9–11]. Therefore, the prevalence of PI between young and old patients with PACG was also compared in the present study. The main purpose of this study was to compare the anatomical characteristics and pathogenesis of angle closure between young and old PACG patients.

Malignant glaucoma (MG, also known as aqueous misdirection syndrome) is a serious complication, which classically occurs in PACG eyes after trabeculectomy [12, 13]. Young PACG patients are reported to have a higher incidence of MG after trabeculectomy compared to older PACG patients [14–16]. In the present study, the ocular structural characteristics of patients who developed MG after trabeculectomy were also evaluated. In addition, the probability of the occurrence of MG after surgery was determined and the biometric predictors for the development of MG in young patients with PACG were assessed.

## Materials and methods

### Patients and data collection

This study was a retrospective, comparative study. We searched our database for patients who were diagnosed with acute or chronic PACG and received UBM examination in Xiangya Hospital,Central South University from January 1, 2016 to January 1, 2020. The study was conducted in accordance with the ethical principles specified in the Declaration of Helsinki and was approved by the Xiangya Ethics Committee.

PACG was defined as the presence of appositional angle closure over 270° or more (posterior trabecular meshwork not visible during static gonioscopy in more than three-quarters of the angle circumference, in primary gaze), IOP > 21 mm Hg, and corresponding glaucomatous optic neuropathy. Exclusion criteria were as follows: 1) previous intraocular surgery,including cataract, anti-glaucoma surgery and laser peripheral iridotomy( LPI); 2) patients with lens subluxation or intumescent cataract; 3) patients with uveal effusion; 4) patients with retinal detachment; 5) patients with an AL < 19 mm in either eye. Age 45 was chosen as an arbitrary cut off for defining “young” in this study; patients aged ≤ 45 years old were included in the young group. Patients aged > 45 years old were included in the old group. Because there are too many cases in the old group, we selected some of patients for analysis. In order to reduce the bias, the first ten patients who received UBM examination in our department every month were included.

**Fig. 1:**
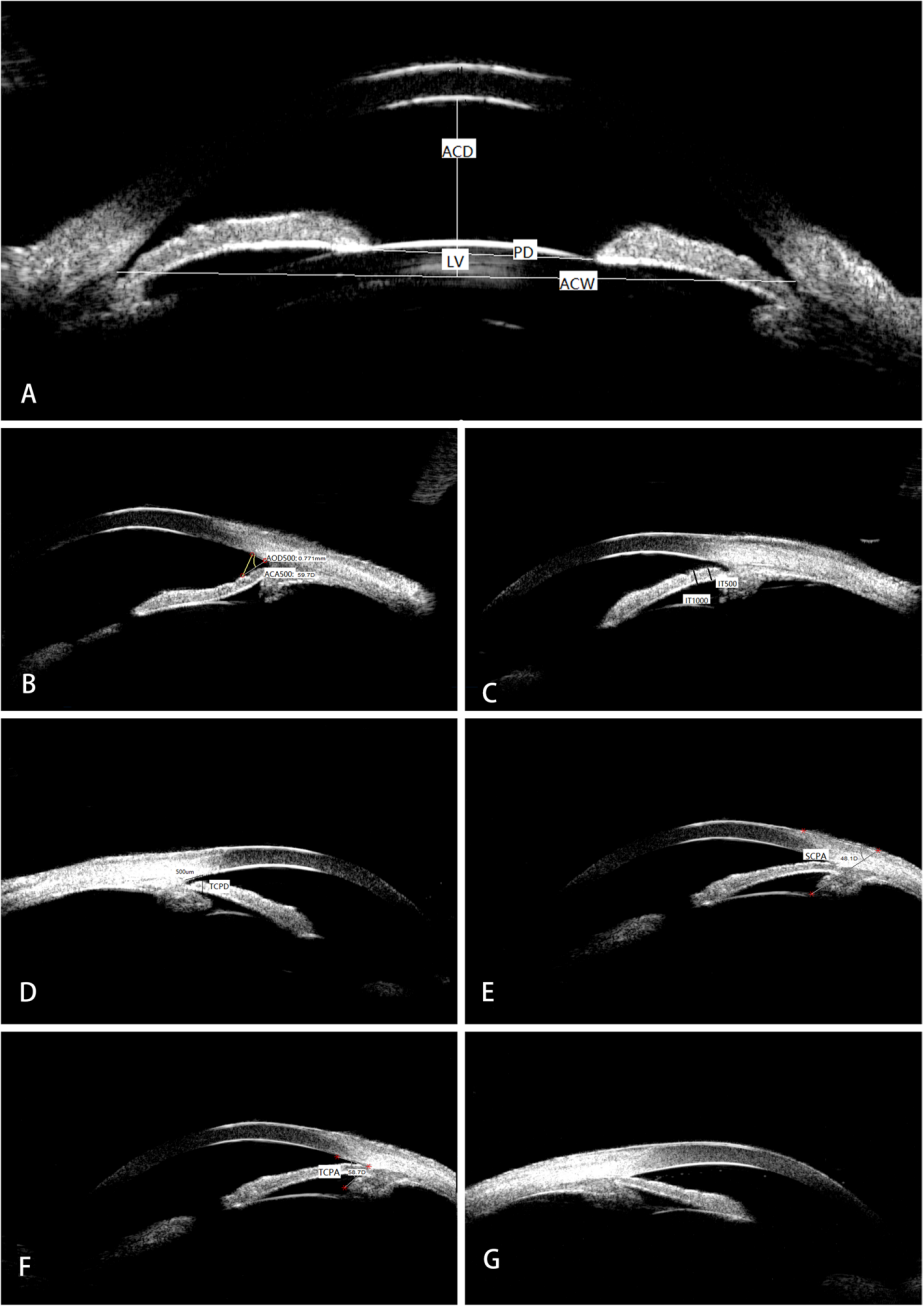
Ultrasound biomicroscopy image showing the measurements of ocular biometric parameters. **A** The measurements of central anterior chamber depth (ACD), pupil diameter (PD), anterior chamber width (ACW), and lens vault (LV). **B** The measurements of anterior chamber angle 500 (ACA500), angle opening distance 500 (AOD500). **C** The measurements of iris thickness at 1000 µm from iris root (IT1000) and iris thickness at 500 µm from iris root (IT500). **D** The measurements of trabecular meshwork-ciliary process distance (TCPD). **E** The measurement of scleral–ciliary process angle (SCPA). **F** The measurement of trabecular meshwork-ciliary process angle (TCPA).** G** Image of a quadrant depicting basal iris insertion

The following demographic and clinical data were extracted from the medical record: age, gender, clinical diagnoses, age at diagnosis of PACG, visual field (VF), and parameters measured by A-scan and UBM. A-scan was used to measure AL and lens thickness (LT). UBM examinations were performed in a supine position in a dimly lit room and the UBM parameters were measured as described previously [17]. ACD, pupil diameter (PD), anterior chamber width (ACW) and lens vault (LV) were measured on horizontal perpendicular scans centered over the pupil. ACD was measured from the corneal endothelium to the anterior lens surface, ACW was measured from the nasal scleral spur to the temporal scleral spur and LV was measured from the anterior pole of the crystalline lens to the horizontal line joining the 2 scleral spurs (Fig. 1). Anterior chamber angle 500 (ACA500), angle opening distance 500 (AOD500). iris thickness 500 μm from the iris root (IT500), iris thickness 1000 μm from the iris root (IT1000), trabecular-ciliary process distance (TCPD), trabecular-ciliary process angle (TCPA), and scleral– ciliary process angle (SCPA) were measured on the radial scans at the 12-, 3-, 6-, and 9-o’clock positions centered over the limbus (Fig. 1). The nasal, temporal, superior, and inferior aspects of these parameters were averaged. ACA500 or the trabecular-iris angle was measured with the apex in the iris recess and the arms of the angle passing through a point on the trabecular meshwork at 500 μm from the scleral spur and the point on the iris perpendicularly opposite. AOD500 refers to the distance between the posterior cornea surface and the anterior iris surface measured on a line perpendicular to the trabecular meshwork 500 μm from the scleral spur; IT500 is the iris thickness at 500 μm from iris root; and IT1000 is the iris thickness 1000 μm from iris root. TCPD was measured as a line extending from a point 500 μm anterior to the scleral spur along the corneal endothelium and dropped perpendicularly through the iris to the most anterior ciliary process seen while scanning in that meridian. SCPA were measured between the line tangent to the scleral surface and the axis of the ciliary process and TCPA was measured with the apex in the scleral spur and the arms of the angle passing through the apex of ciliary process and tangent to the inner surface of cornea. Iris insertion was determined by the location of iris insertion on the ciliary body, and basal iris insertion refers to iris insertion that is located at the base of the ciliary body near the scleral spur (Fig. 1). A-scan ultrasound biometry (Model KN-3000A; Quatel Co Ltd., France) and UBM (Model SW-3200L; Tianjin Suowei Electronic Technology Co Ltd., China) examinations and measurements were performed by the same well-trained physician.

PI was defined based on UBM images using standardized qualitative criteria as described by Kumar et al., that is, anteriorly directed ciliary process, absent ciliary sulcus, steep iris root from the point of insertion followed by a downward angulation, flat iris plane, and irido-angle contact (above the level of the scleral spur) in the same quadrant. At least 2 quadrants had to fulfill these UBM criteria to be defined as having PI [11].

We observed the incidence of MG after trabeculectomy surgery in the two groups. Patients who received cataract surgery and trabeculectomy at the same time were excluded. The diagnosis of MG was established based on the following: i) presence of a central and peripheral shallow or flat anterior chamber with a patent iridectomy; and ii) elevated IOP (≥ 21 mmHg). Eyes with suprachoroidal hemorrhage and postoperative bleb leakage were not considered as MG in our study. To confirm whether patients had postoperative MG, all medical records for each patients were checked, and follow-up visits were conducted.

Only one eye per patient was analyzed in this study. If both eyes were eligible for study, data from the the eye with more serious glaucoma was chosen for analysis. Mean deviation (MD) in visual field was used to evaluate the progress of glaucoma.

### Statistical analysis

Statistical analyses were performed using SPSS statistics software 24 (International Business Machines Corporation, USA). The numerical variables are described as mean ± standard deviation (SD) while the categorical variables are described as numbers and percentages.

Differences in mean values between young and old PACG eyes were examined using the unpaired *t*-test. To determine the possible factors affecting the occurrence of malignant glaucoma in young PACG patients, all the variables measured by A-scan and UBM were assessed using univariate logistic regression analysis. Variables with *P* < 0.1 in univariate logistic regression analysis were included in the multivariate logistic regression model. Consequently, insignificant factors were removed using a stepwise approach. *P* < 0.05 was considered statistically significant.

## Results

From January 1, 2016 to January 1, 2020, 1220 patients were diagnosed with acute or chronic PACG in our department and met our inclusion criteria, of which, 115 patients (9.43%) younger than 45 years old were included into the young group and 480 patients older than 45 years old were included into the old group. The clinical data of young and old PACG patients were shown in Table 1. There were 83 (72.2%) female patients in the young group and 287(59.8%) female patients in the old group (*P* = 0.02). No significant difference in visual field MD or PSD was found between the two groups (*P* > 0.05).

**Table 1:** Comparison of the demographic and clinical characteristics of the young and old PACG patients

Characteristic	Young group	Old group	*P*
No. of patients	115	480	
Female/male	83/32	287/193	0.02
Age	39.2 ± 6.7	61.8 ± 8.2	< 0.001
MD of VF (dB)	− 14.0 ± 6.5	− 15.5 ± 5.3	0.78
PSD of VF (dB)	6.9 ± 4.1	7.0 ± 3.1	0.67

*PACG* primary angle-closure glaucoma, *MD* mean deviation, *PSD* pattern SD, *VF* visual field

A-scan and UBM parameters in the young and the old group were shown in Table 2. TCPD and AL were significantly shorter, TCPA and SCPA were significantly narrower, and LT was significnatly thinner in the young group compared to those in the old group. However, there were no significant differences in ACD, AOD 500, ACA500, LV, IT500, IT1000, PD and ACW between the two groups. The prevalence of PI was 22.5% in the old group while was 66.1% in the young group (*P* < 0.001). Basal iris insertion was found in 72 (62.6%) patients in the young group and 96 (20.0%) patients in the old group (*P* < 0.001).

**Table 2:** Comparison of ocular biometric parameters in young and old PACG patients

Parameter	Young group	Old group	*P*
AL (mm)	21.78 ± 1.00	22.68 ± 0.87	< 0.001
LT (mm)	4.22 ± 0.69	4.56 ± 0.64	0.03
AOD500 (mm)	0.02 ± 0.04	0.02 ± 0.03	0.87
ACA500 (Deg.)	2.70 ± 4.84	2.82 ± 2.4	0.76
TCPA (Deg.)	52.87 ± 11.34	63.28 ± 18.41	< 0.001
TCPD (μm)	0.44 ± 0.08	0.51 ± 0.09	< 0.001
SCPA (Deg.)	61.32 ± 11.68	73.59 ± 11.85	< 0.001
IT500 (μm)	0.36 ± 0.06	0.37 ± 0.06	0.50
IT1000 (μm)	0.43 ± 0.07	0.44 ± 0.06	0.58
ACD (μm)	2.01 ± 0.40	1.91 ± 0.23	0.16
LV (mm)	0.84 ± 0.26	0.95 ± 0.50	0.22
PD	3.19 ± 0.89	3.41 ± 0.95	0.17
ACW	11.55 ± 0.74	11.33 ± 0.73	0.20
PI (No)	76 (66.1%)	108 (22.5%)	< 0.001
BII (No)	72 (62.6%)	96 (20.0%)	< 0.001
PMG (No)	21(24.1%)	11 (5.5%)	< 0.001

*AL* axial length, *LT* lens thickness, *AOD500* angle opening distance 500, *ACA500* angle chamber angle 500, *TCPA* trabecular-ciliary process angle, *TCPD* trabecular-ciliary process distance, *SCPA* scleral– ciliary process angle, *IT500* iris thickness at 500 μm from iris root, *IT1000* iris thickness at 1000 μm from iris root, *ACD* central anterior chamber depth, *LV* lens vault, *PD* pupil diameter, *ACW* anterior chamber width, *PI* plateau iris, *BII* basal iris insertion, *PMG* postoperative malignant glaucoma

Besides, 24 young patients and 164 old patients received medical treatment, 4 young patients and 115 old patients received combined surgery with glaucoma and cataract. There were 87 young patients and 201 old patients underwent trabeculectomy in our study. Among these patients, 21 young patients (24.1%)and 11 old patients (5.5%)developed MG after trabeculectomy (*P* < 0.001). Table 3 listed the relevant variables that predict the occurrence of MG in young patients with PACG using multivariate regression with the Generalized Estimation Equation. TCPD, TCPA and AL were associated with the occurrence of MG in young patients (*P* < 0.05).

**Table 3:** Factors predicting the development of PMG in young patients with PACG

Factor	*β*	*SE*	95% CI		Hypothesis Test		
			**Lower**	**Upper**	**Wald Chi-Square**	**df**	***P***
TCPA	− 1.12	0.54	0.11	0.88	4.80	1	0.03
TCPD	168.85	74.93	3,586,933,959	1.28E + 137	5.08	1	0.02
AL	− 2.36	0.86	0.02	0.54	7.63	1	0.006

*AL* axial length, *CI* Confidence Interval, *PMG* postoperative malignant glaucoma, *PACG* chronic primary angle-closure glaucoma, *SE* standard error, *TCPA* trabecular-ciliary process angle, *TCPD* trabecular-ciliary process distance

## Discussion

In the present study, ocular biometric parameters including AL, LT, ACD, LV, PD, ACW, TCPA, TCPD, SCPA, LT, IT500, IT1000, AOD500, and ACA 500 were quantitatively compared between young and old PACG patients. Among these ocular biometric parameters, shorter TCPD and AL, narrower SCPA and TCPA, thinner LT were found in young PACG patients when compared to old PACG patients. To the best of our knowledge, this is the first study detailing the ocular biometric characteristics of young patients with PACG.

TCPD, TCPA and SCPA are regarded as important parameters that reflect the anterior position of ciliary processes [11, 18, 19]. Narrower SCPA and TCPA and shorter TCPD indicate a more anterior position of ciliary processes. The anterior rotation of ciliary processes would push the iris root forward, resulting in angle narrowing, which could be a predisposing factor for the development of creeping angle closure. In our study, SCPA and TCPA were narrower and TCPD was shorter in young PACG patients than old PACG patients, indicating a more anterior position of ciliary processes is a predisposing factor for the etiology of PACG in young patients.

Several previous studies have suggested that the iris play an important role in the pathogenesis of angle closure [8, 20, 21]. A thicker peripheral iris and a more basal iris insertion were associated with an increased risk of angle closure. In our study, peripheral iris thickness was similar between the old and young PACG patients, but basal iris insertion was more common in the young PACG patients than in the old PACG patients.

The thicker and more anteriorly located lens, as well as a shorter AL, are thought to be predisposing factors for angle-closure [22–25]. In our study, we also found that higher LV and thicker LT in the old patients. LT and LV will increase with age, and this factor may contribute to the increase in incidence of angle closure with age [21].Compared to the old patients with PACG, young PACG patients displayed shorter AL,we hypothesised that the natural anatomical abnormality with short AL was a cause of angle closure in these patients.

Pathogenesis of angle closure glaucoma can be divided into pupillary block mechanism and non-pupillary block mechanism. Although pupillary block mechanisms are the main cause of angle closure in PACG patients [7], non-pupillary block,especially PI, is an important pathogenesis of angle closure in young patients. Ritch et al. [5] systematically examined the demographics and clinical appearance of young individuals with angle closure and found that PI was the most common diagnosis (52.2%) in a relatively inhomogeneous sample of 67 patients with angle closure symptoms. Previous studies also showed that the prevalence of PI in primary angle-closure suspect (PACS) eyes was about 30% in Asian people, and patients with PI tend to be younger [10, 12]. In PI, large and anteriorly inserted ciliary processes hold the iris root in apposition to the trabecular meshwork, resulting in spontaneous or provoked acute or intermittent angle closure. In this study, we specificly compared the incidence of PI between young and old PACG patients and found that the prevalence of PI was 66.1% in the young patients with PACG, which was significantly higher than that in the old patients. Our results indicated that in contrast to old PACG patients, PI was the most common underlying etiology in young PACG patients.

MG is typically known to occur after glaucoma filtration surgery in eyes with PACG and the incidence of MG has been reported to be approximately 0.6% to 4% [12]. Young age is an important risk factor for the development of MG. In our study, the incidence of postoperative MG was 24.1% in young patients with PACG (Table 3), which was much higher than that in the old patients, and higher than those reported in previous studies [12]. To our knownedge, this was the first time that reported the incidence of MG in young patients with PACG after glaucoma filtration surgery. Moreover, we found that shorter AL and more anteriorly rotated ciliary bodies might be predictors for the development of MG in young patients with PACG.

Our study was limited by its retrospective design, which may have affected the selection of patients. Another limitation of this study was that the gonioscopic and quantitative measurements of UBM images were performed by the same examiner, which could cause observational bias.The third limitation of this study was lack of normative data since the ocular parameters such as LT and LV will change with age. All the patients in our study were Chinese, so the results may not be generalizable to other ethnicities.

In conclusion, compared to old patients with PACG, young patients with PACG had shorter ALs, more anterior position of ciliary processes, more cases of basal iris insertion, higher prevalence of PI and higher incidence of MG. These anatomic abnormalities could be the reason that young patients develop angle closure at much earlier age and develop MG more easily after trabeculectomy. In contrast to old PACG patients, non-pupil block mechanisms are the main cause of angle closure in young patients with PACG, thus iridectomy may not be an effective treatment for angle closure in these patients [26]. Therefore, in young patients with glaucoma, gonioscopy and UBM should be used to examine the angle structure, to determine the pathogenesis of glaucoma and provide corresponding treatment.

## Data Availability

The datasets used during the current study are available from the corresponding author on reasonable request.
